# Strategies to reduce the risk of falling: Cohort study analysis with 1-year follow-up in community dwelling older adults

**DOI:** 10.1186/s12877-016-0267-5

**Published:** 2016-04-29

**Authors:** John N. Morris, Elizabeth P. Howard, Knight Steel, Katherine Berg, Achille Tchalla, Amy Munankarmi, Daniel David

**Affiliations:** Hebrew SeniorLife, Institute for Aging Research, 1200 Centre Street, Boston, MA 02131 USA; School of Nursing, Northeastern University, 360 Huntington Avenue, Boston, MA 02115 USA; Hackensack University Medical Center, 30 Prospect Avenue, Hackensack, NJ 07601 USA; Department of Physical Therapy and Rehabilitation Sciences Institute, University of Toronto, 160-500 University Avenue, Toronto, Ontario M5G 1V7 Canada; Geriatric Medicine Department, Limoges University, IFR 145 Geist; EA 6310 HAVAE, CHU Limoges, Limoges, F-87025 France; Northeastern University, 360 Huntington Ave, Boston, MA 02115 USA; VA Quality Scholar, Geriatrics, Palliative and Extended Care Service, San Francisco Veterans Affairs Medical Center, San Francisco, CA 94121 USA

**Keywords:** Strategies to reduce risk of falls, Community-dwelling older adults, interRAI assessment

## Abstract

**Background:**

According to the CDC, falls rank among the leading causes of accidental death in the United States, resulting in significant health care costs annually. In this paper we present information about everyday lifestyle decisions of the older adult that may help reduce the risk of falling. We pursued two lines of inquiry: first, we identify and then test known mutable fall risk factors and ask how the resolution of such problems correlates with changes in fall rates. Second, we identify a series of everyday lifestyle options that persons may follow and then ask, does such engagement (e.g., engagement in exercise programs) lessen the older adult’s risk of falling and if it does, will the relationship hold as the count of risk factors increases?

**Methods:**

Using a secondary analysis of lifestyle choices and risk changes that may explain fall rates over one year, we drew on a data set of 13,623 community residing elders in independent housing sites from 24 US states. All older adults were assessed at baseline, and a subset assessed one year later (*n* = 4,563) using two interRAI tools: the interRAI Community Health Assessment and interRAI Wellness Assessment.

**Results:**

For the vast majority of risk measures, problem resolution is followed by lower rate of falls. This is true for physical measures such as doing housework, meal preparation, unsteady gait, transferring, and dressing the lower body. Similarly, this pattern is observed for clinical measures such as depression, memory, vision, dizziness, and fatigue. Among the older adults who had a falls risk at the baseline assessment, about 20 % improve, that is, they had a decreased falls rate when the problem risk improved. This outcome suggests that improvement of physical or clinical states potentially may result in a decreased falls rate. Additionally, physical exercise and cognitive activities are associated with a lower rate of falls.

**Conclusions:**

The resolution of risk problems and physical and cognitive lifestyle choices are related to lower fall rates in elders in the community. The results presented here point to specific areas, that when targeted, may reduce the risk of falls. In addition, when there is problem resolution for specific clinical conditions, a decreased risk for falls also may occur.

## Background

Falls are common among older persons and rank among the leading causes of accidental death in the United States, resulting in significant health care costs annually [1, 2]. Falls account for approximately 10 % of visits to an emergency department and 6 % of hospitalizations among Medicare beneficiaries [3]. For many, falls are an everyday risk as the person walks about the home, climbs a flight of stairs, or performs normal daily activities [2]. For a few, a fear of falling can limit their going outdoors or even walking in their own home [4–6]. In short, to be human is to be at risk of falling and it is crucial that we increase our awareness of strategies that have the potential of lessening the older adult’s risk of falling. Presently, an estimated 33 % of older adults fall each year and this rate rises to nearly 50 % for persons over the age of 80 [7].

At the same time, most falls have little lasting consequence on a person’s ongoing daily life: no injury results, no bones are broken, and the person continues to engage in activities with no obvious fear of falling [8]. The elder may stumble rising from bed, trip on an uneven surface, or slip on a loose rug, and when these things occur, the person gets up and goes on with life. For many elders, falls become an acceptable part of life.

For a small percentage, however, there are more serious consequences of having fallen, including bruises, broken bones, and even death [9]. The overall annual rate of falls for elders 65–74 is 57 per 1,000 and for those 75 and older, this rate increases to 115 per 1000 [10]. While some falls may just be unlucky one-time events, for others the fall itself is a signal that the person has underlying conditions that predispose the person to further falls [11, 12].

Our goal in this paper is to better understand how the everyday lifestyle decisions of the older adult may help reduce the risk of falling. To accomplish this goal, we focus on two lines of inquiry. First, we identify and then test known mutable fall risk factors and ask how the resolution of such problems correlates with changes in fall rates. Second, we identify a series of everyday lifestyle options that persons may follow and then ask, does such engagement (e.g., engagement in exercise programs) lessen the older adult’s risk of falling and if it does, wiill the relationship hold as the count of risk factors increases?

### Factors influencing falls

A sizeable body of work has identified independent factors that increase one’s risk of falling, not all of which are susceptible to change. In our work we concentrate only on risk factors that have the possibility of improving over time. This is not to say that immutable factors are not relevant, for they are, but for this research we focus on potentially changeable risk factors. We organize these into two broad domains: physical performance and clinical complexity. Table 1 lists the specific factors and also provides the assessment items used to examine these two domains.

**Table 1:** Fall risk domain and assessment item

Fall risk domain	Assessment item
Physical –Movement Related	Unsteady gait
	Walks with help
	Climb stairs with help
	Requires help in doing housework
	Requires help in cooking
	Requires help in shopping
Physical –Weakness/Debility	Dressing lower body with help
	Transfers with help
	Bathes with help
	Experienced worsening ADL in 90 days
Clinical – Cognitive/Mental	Cognitive decline in past 90 days
	Memory problem
	Anxiety diagnosis
	Depression diagnosis
Clinical -- Physical/General	Vision problem
	Dizziness
	Poor self-reported health
	Not continent
	Acute health flare-up
	Constipation (tested, not significant)
	Difficulty sleeping (tested, not significant)
	Fatigue
	Pain intensity
	Breakthrough pain

These factors incorporate the areas of functional ability, cognition, and health conditions. Elders who need assistance with movement such as walking, transferring from bed to chair, and stair climbing are at greater risk for falling [13–15]. Use of assistive devices for ambulation such as a cane or walker is associated with an increased fall risk as is diminished functional ability [16–19]. Elders dependent in activities of daily living (ADLs) had an increased fall risk and an increased incidence of falls [17, 19]. Stenhagen (2013) reported dependence in one or more ADLs as a falls risk, with an age-adjusted odds ratio (OR) of 1.49 [20]. Dependence in instrumental activities of daily living (IADLs) produced similar results [20–22].

Cognitive impairment also was associated with an increased fall risk [23, 24]. Fischer (2014) found cognitively impaired elders had an increased risk of falls and raised the issue of whether cognitively impaired elders engage in risky activities, thereby increasing their fall rate [25]. Diminished scores in executive function and attention were associated with future fall risk [26]. Furthermore, multiple falls and increased falls risk are seen in the presence of executive and visuo-spatial function deficits [24, 27].

There is a wide spectrum of health conditions associated with an increased fall risk and a history of falls among the elderly. An unsteady gait and a self-report of dizziness are two symptoms reasonably associated with an increased fall risk [20, 21, 28–31]. Both call into question the presence of vestibular impairment. Self-reported balance impairment was a significant predictor of future falls [32, 33]. Visual impairment inclusive of poor gaze stabilization, decreased acuity and incorrect use of multi-focal lens in glasses all contribute to a history of falls and increased fall risk [34–36]. Dyspnea, often resulting from COPD or heart failure, in older adults also is predictive of future falls [20, 37]. Several researchers also found obesity associated with increased risk of falls [11, 38, 39].

### Protective lifestyle choices

The second focus of this report is on everyday lifestyle choices that older adults may adopt to potentially reduce their risk of falling, including those choices that have physical and cognitive foundations. Reports in the literature support an association between falls and early lifestyle physical and cognitive enhancement choices. Group and home-based exercise programs reduced both falls rate and the risk of falling. Tai Chi reduced the risk of falling [40]. Moreira et al. found high-intensity aquatic exercises reduced the number of falls among post-menopausal women [41]. In another study, Yamada, et al. reported that a 24 week program of rhythmic stepping exercise decreased the relative risk of fear of falling [42].

There is limited information about falls and specific cognitively-stimulating activities, such as crossword puzzles and computer use. The use of a computer assisted technology, such as the Nintendo Wii, provides both visual training and assistance with obstacle avoidance and, as such, may have value as a fall prevention intervention [43]. There is a growing body of work on the association between cognitive training and improvement with gait and balance that impacts both the number of falls and fall rates [44, 45].

Table 2 lists the measures assessed using the interRAI personal self-report Wellness Tool and, while not matched precisely with activities examined in prior research, it provides an adequate representation of the 2 domains of lifestyle choices associated with improving fall risk and occurrence [46].

**Table 2:** Domains protective of falls risk

Protective domains	Assessment item
Physical Exercise	Three + hours exercise a day
	Bikes
	Hikes/walks
	Swims
	Pilates/yoga/Tai Chi
	Treadmill/steppers/weights/resistance
Cognitive Stimulation	Uses computer
	Does crosswords
	Takes education course(s)

## Methods

The site for this project was COLLAGE, a national consortium of CCRCs and elder housing sites initiated and developed by Kendal Outreach, LCC (KOLCC), a subsidiary of the Kendal Corporation, a non-profit organization, and the Institute for Aging Research (IFAR) at Hebrew SeniorLife, a Massachusetts non-profit corporation. Members of the consortium participated in the application of the first, computerized, valid and reliable approach to annual standardized resident assessments in the US within senior housing [15]. Key staff of senior housing sites that are members of the COLLAGE consortium meet with residents, and through a semi-structured conversation, complete two assessment tools, the Community Health Assessment (CHA) and the Wellness Tool (WELL). The CHA was developed by interRAI [16], a collaborative network of researchers in over 35 countries committed to improving health care for persons who are elderly, frail, or disabled. InterRAI’s goal is to promote evidence-based clinical practice and policy decisions through the collection and interpretation of high quality data about the characteristics and outcomes of persons served across a variety of health and social service settings. The WELL was developed collaboratively by COLLAGE and interRAI [17]. These assessment tools have been used both nationally and internationally, and represent the results of rigorous research and testing to establish the reliability and validity of their items [17]. They move beyond the typical health assessment and physical examinations routinely completed at a health care visit to provide a holistic summary of the physical, psychosocial and environmental elements impacting an elder’s quality of life.

Using a secondary analysis of risk changes and lifestyle choices that may help to explain fall rates over just one year, we draw on a data set that includes 13,623 community residing elders in independent housing sites in 24 US states. All of these older adults participate in the COLLAGE program (now known as Vitalize 360), and were assessed at baseline with a subset assessed one year later (*n* = 4,563) using two interRAI tools: the interRAI Community Health Assessment and interRAI Wellness Assessment [47]. Both studies were approved by the Hebrew SeniorLife, Institute for Aging Research, Institutional Review Board (IRB).

### Availability of data and materials

The COLLAGE consortium contracted with interRAI to use the data collection tools to complete the comprehensive assessments. InterRAI receives no royalties for use but requires all data be sent to their international repository. De-identified research copies of the data are maintained by interRAI with an understanding the data may be used for research purposes only by individuals and organizations who have a direct affiliation with interRAI.

Assessors trained in the use of the interRAI instruments completed all assessments. The training occurred at each site and followed models specified by interRAI [47]. Therefore, the reliability of the available data elements can be presumed to be consistent with data reported previously [46, 48, 49].

Each person is questioned about their falls over the 90-day period prior to the assessment, and these measures were used as dependent variables in this work.

### Analytic approach

The analyses of the role of the baseline falls risk factors involve a comparison of the best dichotomous version of each measure against the falls dependent variables. The relationship is tested for significance (based on a .05 or lower two-tailed probability for chi-square) and association (odds ratio). Where significant, the uni-variate odds ratio (OR) is presented to gage the strength of the relationship.

The same approach was employed to assess the role of protective lifestyle choices selected by the older adults. As a second part of this lifestyle analysis, a summary risk scale was constructed, summing the earlier risk measures. Stepwise logistic regression was used to reduce the previously assessed risk measure pool prior to those variables being summed into a fall risk index. The baseline fall rates at each level of the risk index scale are described, as is the variation in these rates at each risk level as a function of the protective lifestyle options.

## Results

The older adults in the independent housing sample (COLLAGE) came from 24 of the 50 US states and had their baseline assessment carried out between 2004 and 2013. Their average age was 81 years. In terms of basic demographics, 90 % were white, 68 % were female, 49 % were married, and 53 % lived alone.

Functionally and cognitively, most sample members were independent at the time of the baseline assessment. From a functional perspective, 93 % walked without the help of others and 90 % could prepare a meal without assistance. The cognitive picture shows an equally independent population. Eighty-seven percent were independent in decision-making, 80 % had no problem with short-term memory, and 88 % fully understand others with whom they communicated. While 59 % reported they were occasionally forgetful, only 5.8 % described their memory as being a problem.

In terms of falls, 13.8 % reported a fall in the 90 days prior to the baseline assessment. At follow-up, the percentage haven fallen in previous 90 days was the same: 13.8 %. Among those who had fallen in the 90-days prior to the baseline assessment, 25 % indicated that a fear of falling limited their going outdoors. This contrasts with 9.8 % for those who had not fallen in this period. A similar pattern emerges for those who say that a fear of falling caused them concern when walking in their home: 24.9 % vs. 9 %.

### Falls and selected baseline characteristics

**Fig. 1:**
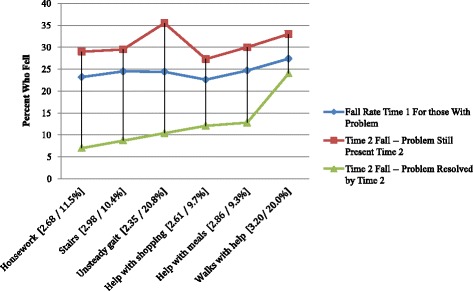
Fall Rates Based on Movement Related Risk Variables (with time 2 risk odds ratios and % problem resolved)

**Fig. 2:**
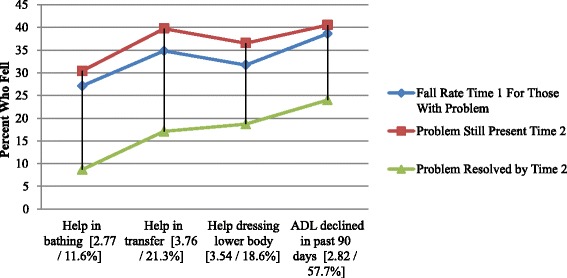
Fall Rates Based on Weakness/Debility Related Risk Variables (with Time 2 risk odds ratios and % resolved)

We first examined the baseline physical factors (Figs. 1 and 2) that might contribute to falls. All six movement-related independent variables were significantly related to falls at times 1 and 2 – the time 2 odds ratios are given in brackets on Fig. 1. These odds ratios are all high: ranging from 2.3 to 3.2 at time 2 (the follow-up assessment). Five of the measures also meet the criterion of a sharp drop in fall rates with problem resolution by time 2. The exception was “walks with help”.

In Fig. 2 we see a similar pattern for all four weakness/debility physical risk measures. All are significantly related to falls at times 1 and 2. The time 2 odds ratios are all high: ranging from 2.7 to 3.7. All four of the measures also meet the criterion of a sharp drop in fall rates with problem resolution by time 2.

**Fig. 3:**
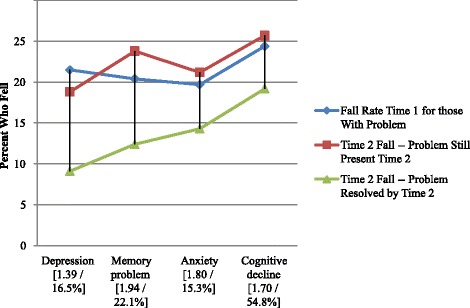
Fall Rates Based on Cognitive/Mental Related Risk Variables (with Time 2 risk odds ratios and % resolved)

Next we examine the baseline clinical complexity measures that might contribute to falls. Figure 3 displays information for the cognition and mental health measures. All are significantly related to falls at times 1 and 2 – in this case the time 2 odds ratios are less than those seen initially, all falling below 2.0. The measures of depression and memory problem meet the criterion of a sharp drop in fall rates with problem resolution by time 2. The other two measures drop at a somewhat lower rate.

**Fig. 4:**
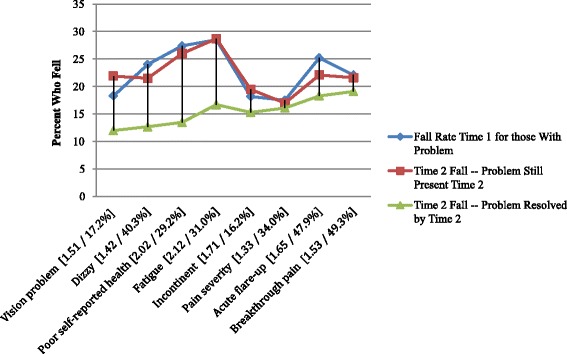
Fall Rates Based on Physical and General Clinical Complexity Related Risk Variables (with time 2 risk odds ratio and % resolved)

Figure 4 displays the results for the physical and more general measures of clinical complexity. Two of the measures are not displayed because baseline problems in these areas were not related to time 2 fall rates. These omitted measures were constipation and difficulty sleeping. Of the remaining eight measures, all are significantly related to falls at times 1 and 2, but only four of the measures meet the criterion of a sharp drop in fall rates with problem resolution by time 2 [vision, dizziness, poor self-reported health, and fatigue].

### Lifestyle choices related to falls

**Fig. 5:**
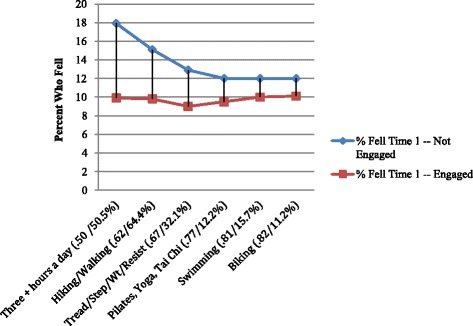
Time 1 Fall Rates Based on Types of Exercise at Baseline (with time 1 risk odds ratio and % so engaged)

Figure 5 displays the results for measures of physical exercise vs. time 1 and time 2 fall rates. All rates are significant and, when the person is engaged in the exercise, the fall rate drops to a little less than 10 %. Half of all members of the cohort were engaged in three or more hours of exercise a day, and for those not so involved, we see the highest rate of falls (17.9 % -- or almost twice the rate of those who were engaged in this level of exercise).

**Fig. 6:**
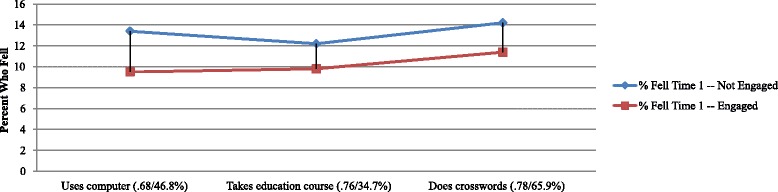
Time 1 Fall Rates Based on Engagement in Cognitively Stimulating Exercises (with time 1 risk odds ratio and % so engaged)

Figure 6 displays the results for engagement in cognitively stimulating activities. In all three instances, cognitive engagement is associated with lower rates of falls.

**Fig. 7:**
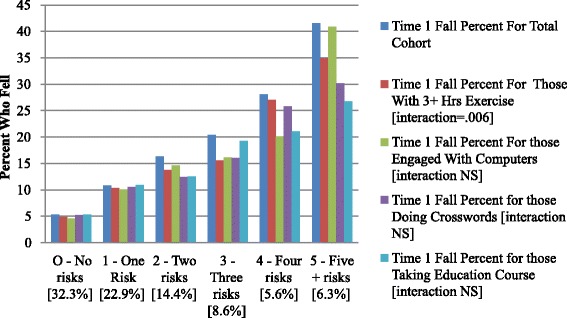
Time 1 Fall Rates Based on Exercise and Cognitive Stimulation Life Choices Across Categories of Risk Summary Scale (percent of sample in risk category)

Figure 7 gathered the prior findings, assessing whether those with an increased risk of falling benefit equally from engagement in physical exercise and cognitively stimulating activities. The risk measure coming out of the forward stepwise regression based on the risk items is described earlier and includes eight measures [unsteady, stairs, ADL decline, bathing, memory, dizziness, and pain symptoms]. As displayed in Fig. 7, all persons with a risk count of 5 or more are collapsed into the same category.

This analysis indicates that physical exercise and cognitive stimulation do not translate into lower fall rates for those with no or only one risk – the best 55 % of the older adults in the cohort. At all other risk levels, there is a reduction in falls for those who engage in these types of activities.

## Discussion

In an attempt to better understand the complex nature of falls among older adults, we pursued two avenues. First we identified and tested known mutable fall risk factors and examined if resolution of the problem resulted in a decreased fall rate. The second approach examined lifestyle options and if engagement lessens an older adult’s fall risk across the span of fall risk counts. For the physical characteristics associated with movement, problem resolution was associated with a decreased rate of falls. The one exception was the item, walks with help. Among the weakness and debility physical elements, with problem resolution, fall rates dropped sharply. For the cognitive and mental clinical complexity measures, we observed a drop in fall rate only with improvement of memory problems or depression. Only 4 of the general clinical measures, when resolved, resulted in a decreased fall rate. These measures were vision problems, dizziness, poor self-reported health and fatigue.

Examination of lifestyle options focused on two domain, physical exercise and cognitive stimulation. The highest falls rate was among individual did not engage in physical exercise and all the assessed physical exercise items were associated with a decrease in falls rate. The physical measures included a wide variety of tasks: doing three or more hours of exercise a day, walking, using a treadmill, or doing Pilates. These results coincide with the work by Moreira et al. and Yamada et al. who examined the relationship between falls and aquatic exercise and rhythmis stepping exercises respectively [40, 41]. Additionally, participation in cognitive stimulating activities was protective of falls risk. The three cognitive lifestyle options all followed the model, including using a computer, doing crosswords, and taking an education course. These results align with evidence on the association between cognitive training and decreased falls [44]. Expanding involvement in these areas should be considered as a measure for reducing the incidence of falls. The results here indicated pursuit of lifestyle options is most beneficial for older adults with 2 or more falls risk factors.

Over a one-year period a distinguishing characteristic is the uniformity and strength of the relationships for the measures tested. For the vast majority of risk measures, problem resolution is followed by lower rate of falls. This is true for such physical measures as doing housework, meal preparation, unsteady gait, transfer, and dressing the lower body. Similarly this pattern is observed for such clinical measures as depression, memory, vision, dizziness, and fatigue. The percent that improved averages about 20 % of those who had problems at baseline and this suggests that the potential exists for extending this phenomenon. The results reported here thus fit into an emerging picture of positive steps an elder can take to reduce the likelihood of falling. For those at risk, there is evidence that engagement in these types of physical and cognitive exercises reduces ones risk of falling.

## Limitations

Many of the assessment items examined in this project were self-report. The assessors who complete the tools through a conversation with the older adult complete an established training program and are skilled in obtaining the most accurate information possible. In the presence of marked cognitive decline, the assessors would ask a caregiver or significant other about the item. We must be aware of the fact that there are many recognized causes for falls and these data support the complexity. At the same time, this research is based on the experiences of a large court of community residing older adults, with an average age of about 80 years. It was not a randomized trial. The findings are supportive of the work of Kelsey, et al. [50] and Ambrose, et al. [51].

## Conclusions

Physical and cognitive engagement lifestyle choices are related to lower fall rates among elders in the community, as are the resolution of risk problems. The outcomes from the secondary analyses point to areas, which, when targeted, may reduce the risk of falls for an individual. In addition, when there is problem resolution for specific clinical conditions, a decreased risk for falls also may occur. The results offer a framework for geriatric care providers to intervene with improving clinical conditions and offering support to engage in lifestyle strategies that will reduce the risk for falls. Given the rate of falls among all older adults and the potential for additional morbidities following a fall, the results presented here offer some guidelines aimed at improving this pervasive problem among the older adult population.

### Ethics approval and consent to participate

Both the baseline and one year follow up studies described in this paper were approved by the Hebrew SeniorLife, Institute for Aging Research, Institutional Review Board (IRB).

### Consent for publication

Not applicable.

### Availability of data and materials

Data used for the secondary analysis in this project is not availablepublicly. De-identified datasets are maintained by interRAI with an understanding the data may be used for research purposes only by individuals and organizations who have a direct affiliation with the interRAI organization.

## Abbreviations

ADL’s: activities of daily living
IADL’s: instrumental activities of daily living
OR: odds ratio
